# Monte Carlo Simulation of Bony Heterogeneity Effects on Dose Profile for Small Irradiation Field in Radiotherapy

**DOI:** 10.1371/journal.pone.0010466

**Published:** 2010-05-03

**Authors:** Simone C. Cardoso, Victor Gabriel L. Alves, Luiz Antonio R. da Rosa, Luciana T. Campos, Delano V. S. Batista, Alessandro Facure

**Affiliations:** 1 Instituto de Física, Universidade Federal do Rio de Janeiro, Rio de Janeiro, Brasil; 2 Instituto de Radioproteção e Dosimetria, IRD/CNEN, Rio de Janeiro, Brasil; 3 Instituto Nacional de Câncer (INCA), Rio de Janeiro, Brasil; 4 Comissão Nacional de Energia Nuclear, Rio de Janeiro, Brasil; German Cancer Research Center, Germany

## Abstract

In the radiotherapy treatment planning of a lesion located in the head region with small field radiation beams, the heterogeneity corrections play an important role. In this work, we investigated the influence of a bony heterogeneity on dose profile inside a soft tissue phantom containing a bony material. PDD curves were obtained by simulation using the Monte Carlo code EGSnrc and employing Eclipse® treatment planning system algorithms (Batho, Modified Batho, Equivalent TAR and Anisotropic Analytic Algorithm) for a 15 MV photon beam and field sizes of 2×2 and 10×10 cm^2^. The Equivalent TAR method exhibited better agreement with Monte Carlo simulations for the 2×2 cm^2^ field size. The magnitude of the effect on PDD due to the bony heterogeneity for 1×1, 2×2 and 10×10 cm^2^ field sizes increases to 10, 5 and 3%, respectively.

## Introduction

The impact of the inclusion of heterogeneity corrections for new techniques in radiotherapy, such as the intensity modulated radiation therapy and radiosurgery, can be more complex than for conventional 3D planning, partly due to the synergistic effect of many small fields, the presence of heterogeneity and the presence of steep fluence gradients [1].

Dosimetric measurements of small field radiation beams are difficult. The narrow dimensions and steep fluence gradients of beams require a small size detector with good spatial resolution. Several studies have been conducted concerning the dosimetry of narrow photon beams in a homogeneous medium [2]–[7], but the influence in dose distributions of bone equivalent materials in such beams is the subject of only a few papers [8]–[14].

Rustgi et al (1998) studied the dose perturbation immediately behind aluminium sheets used to simulate high-density tissue heterogeneities, such as bone, in circular beams of diameter ranging from 12.5 to 40.0 mm. The perturbation was measured with a diamond detector for a 6 MV photon beam and results were compared with EGS4 Monte Carlo calculations. Cheung et al(2001) verified the accuracy of the dose planning system Leksell GammaPlan using EGS4 Monte Carlo code for standard collimator sizes (4, 8, 14 and 18 mm) and a Co-60 photon beam.

Spirydovich et al (2006) investigated the absorbed dose distribution inside a solid water phantom with embedded high density material irradiated by a 6 MV photon beam of field size 10×10 cm^2^. They compared results obtained with radiochromic film, fluence map Monte Carlo method and superposition algorithm.

Ulmer et al (2005) showed a comparison of depth–dose curves obtained using ECLIPSE algorithm with measurements in a bone phantom for 4×4 cm^2^ and 10×10 cm^2^ fields of 6 MV photons.

Fogliata et al (2007) compared many different analytical dose calculation algorithms with Monte Carlo in bone phantoms for high energy photon beams (6 and 15 MV) and for square (13×13 cm^2^) and elongated rectangular (2.8×13 cm^2^) fields.

Carrasco et al (2004 and 2007) compared measurements, Monte Carlo simulations and treatment planning system calculations for 10×10, 5×5, 2×2 and 1×1 cm^2^ for 6, 10 and 18 MV X-rays spectra. For 1×1 cm^2^ irradiation field they measured beam profiles with films, not obtained PDD curves.

None of these studies compared Monte Carlo simulations and treatment planning system calculations considering bony heterogeneity and small field sizes for 15 MV photon beam.

The purpose of this study is to compare PDD curves obtained with Monte Carlo EGSnrc code and calculations using the Eclipse® treatment planning system algorithms (Batho, Modified Batho, equivalent TAR and Anisotropic Analytic Algorithm) for a 15 MV photon beam in two situations: a narrow irradiation field of 2×2 cm^2^ and a conventional irradiation field of 10×10 cm^2^, using a soft tissue phantom containing a bony heterogeneity. The magnitude of the effect of a bony heterogeneity in the PDD was also quantified using Monte Carlo simulations. The 15 MV photon beam was chosen considering the existence of deep tumors and due to the fact that the effect of the bony heterogeneities are more significant at high energies. The information obtained from Monte Carlo simulations allowed the evaluation of the accuracy on delivering the dose at the presence of heterogeneities and the performance of the different Eclipse® heterogeneity correction algorithms in calculating the patient treatment dose.

## Materials and Methods

### Materials

A geometric head phantom was constructed with dimensions similar to those of a head of an adult. The phantom has a cubic regular form in order to facilitate the understanding of the physical processes of radiation interaction with matter. It was constructed through the superposition of acrylic plates, simulating the soft tissue, and PVC plates, simulating bony tissue. In the case of soft tissue, 13 plates with dimensions of 30×30×1 cm^3^ were used. Five 30×30×0.4 cm^3^ PVC plates were employed in the montage of the bony phantom resulting in a total height of 2 cm that was chosen because this is the average thickness of the cortical bone. To assure that the bone heterogeneity would be out of the build up region, the plates were superposed, resulting in an acrylic block of 5 cm height on a PVC block of 2 cm height on an acrylic block of 8 cm height. The complete acrylic system has a height of 13 cm.

### Methods

#### Head phantom validation

The materials were validated through simulated PDDs obtained considering cortical bone against measurements using PVC as bone tissue-mimicking material, considering the same field sizes.

**Figure 1:**
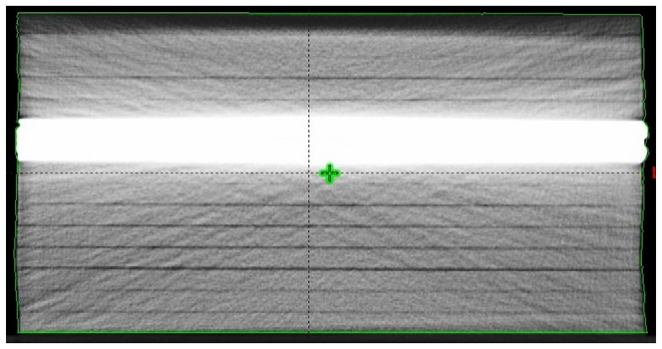
Computerized tomography image of the head phantom. Image used to simulate the soft and bone tissues used in TPS calculations. Five 30×30×0.4 cm^3^ PVC plates and thirteen 30×30×1 cm^3^ acrylic plates were employed in the montage of the soft tissue-bone phantom.

In order to obtain the computerized tomography (CT) images of the head phantom, a PQ2000 CT scanner was used. The equipment belongs to the Brazilian National Institute of Cancer. One hundred tomographic slices (3.0 mm thick) were obtained The high voltage used was 130 kV. Figure 1 shows the image of a computerized tomography of the head phantom used in Treatment Planning System (TPS) calculations.

#### The planning system Eclipse® and the algorithms Batho, modified Batho, equivalent Tissue Air Ratio and Anisotropic Analytic Algorithm

The TPS studied was Eclipse® that calculates the dose with the pencil-beam model and a heterogeneity correction factor by means of four algorithms: Batho, Modified Batho, Equivalent TAR and Anisotropic Analytic Algorithm (AAA) [15]–[19]. All these algorithms were studied for application on the head phantom with bony heterogeneity. The phantom information was introduced by computerized tomography obtained with the PQ2000 CT scanner.

#### Monte Carlo simulation of PDD at the presence of bony heterogeneity

Monte Carlo simulations were performed using the DOSRZnrc [20] user code of EGSnrc [21]. For simulation, a layered phantom consisting of PMMA and cortical bone were created. The PMMA material [22] was used to simulate soft tissue. As in the treatment planning system, the doses were calculated for square field of 2×2 and 10×10 cm^2^ field sizes were transformed in circular ones with the same area generated by a point source located 100 cm far from the surface of the phantom. Incident photons for the Monte Carlo calculations were sampled from the spectrum provided by Mohan [23].

In order to show that the Monte Carlo simulation used the same beam data as the one used by the treatment planning system, a PDD curve in water was simulated using Monte-Carlo code EGSnrc for Mohan spectrum [23]. A field size of 10×10 cm^2^ and a 15 MV photon beam were considered in the simulation. The result was compared to experimental measurements using an ionization chamber carried out with the accelerator Clinac 2300 C/D that is the medical treatment unit used by the treatment planning system.

In order to determine PDD values in a phantom containing a soft tissue equivalent material (PMMA) and a bone equivalent material (PVC), it was necessary to convert dose values for PMMA and bone tissue to values for water, depending on the phantom region considered. This conversion was made on the basis of Bragg-Gray theory [24]. Mass collision stopping power values for cortical bone and PMMA were obtained through simulations using SPRRZnrc from EGSnrc Monte Carlo code.

The global photon energy cutoff value was 1 keV and the cutoff energy for electrons was 521 keV. The number of histories generated was sufficient to produce a statistical variance of less than 0.5% in the dose per incident fluency at the plane of maximum dose for each of the Monte Carlo simulations.

## Results and Discussion

**Figure 2:**
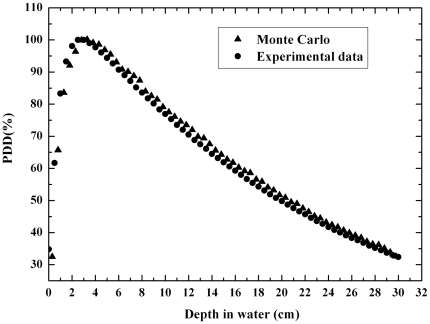
Comparison between PDD curves in water obtained by Monte Carlo simulation and experimentally. The theoretical 15 MV photon spectrum used was determined by Mohan [23].

The spectrum used in this work [23] was validated through the comparison between both PDD curves in water, one simulated using Monte-Carlo code EGSnrc for that spectrum and the other obtained experimentally using an ionization chamber and the 15 MV spectrum generated by the accelerator Clinac 2300 C/D. Results are shown in the figure 2. The deviation between both curves is better than 2%, indicating a good agreement and the adequacy of the chosen theoretical spectrum for the Monte Carlo simulation. If the simulated spectrum had been adjusted to the measured one or full simulations of the linac head had been performed this agreement could be improved.

**Figure 3:**
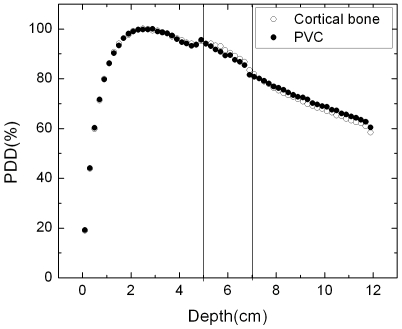
Comparison between PDD curves for PVC and bone. Simulated curves for 15 MV photon beam of 2×2 cm^2^ field size.

The validation of PVC as an appropriate material to simulate the bony tissue was carried out through the comparison between PDD curves simulated with EGSnrc for a soft/bony tissue phantom, considering for the bony tissue region cortical bone and PVC. Results for 2×2 cm^2^ field size are shown in the figure 3. Differences up to 1% were found. These results indicate that PVC can be used as an adequate material to simulate bony tissue for 15 MV photon beam.

The total uncertainty associated to the experimental PDD determination was calculated, through the propagation of specifics uncertainties, as being better than 5%.

Stopping power ratio water-acrylic, water-PVC and water-cortical bone obtained were 0.966, 1.143 and 1.125, respectively.

**Figure 4:**
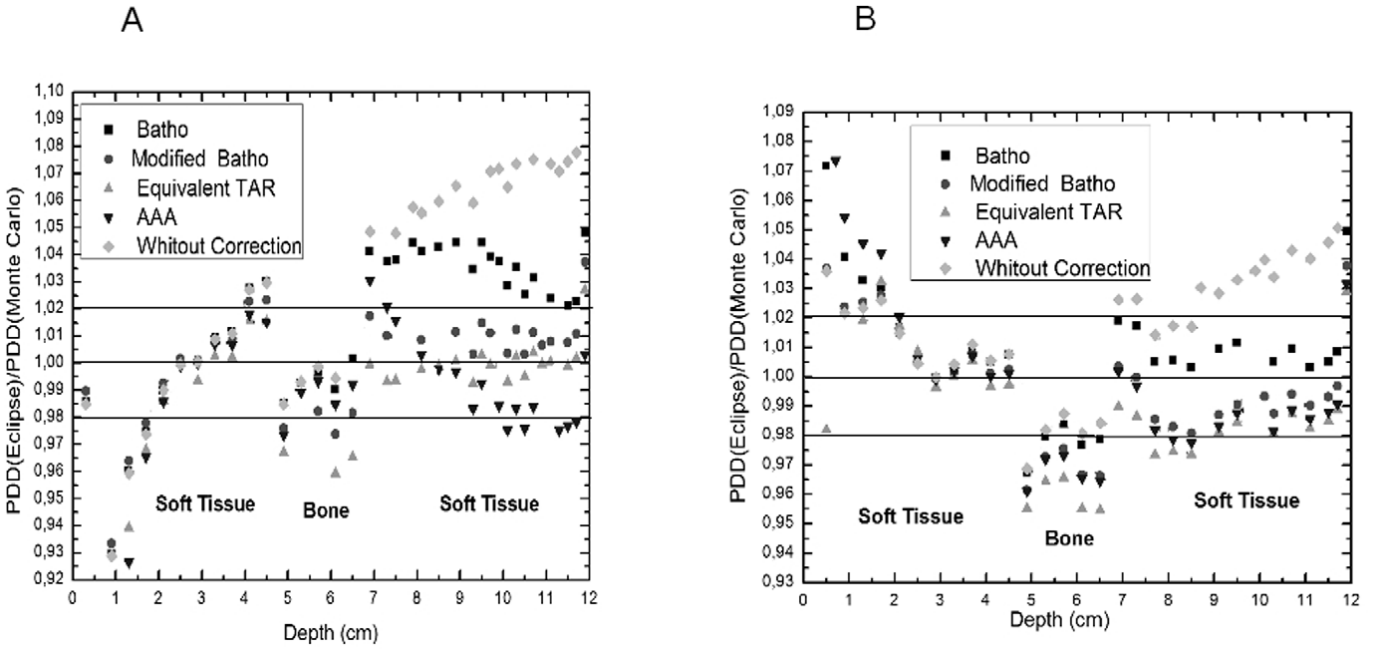
Ratio between calculated and simulated PDD curves. The curves were obtained with the planning system Eclipse and with Monte Carlo simulation (EGSnrc) for (a) 2×2 and (b) 10×10 cm^2^ field sizes.

In order to facilitate the comparison between the PDD curves determined by the planning system Eclipse and by the Monte Carlo code EGSnrc, the ratio between the PDD curves generated by both methods were plotted in the figure 4 for (a) 2×2 and (b)10×10 cm^2^ field sizes.

The figures 4a and 4b show that, for the 2×2 and 10×10 cm^2^ field sizes, differences of the 7% and 4%, respectively, were found after the second interface if no heterogeneity correction method is used. The Batho method presented the biggest difference after the second interface, 4%. This method is not recommended to adjust the dose after a bony heterogeneity for small field sizes. The Equivalent TAR method exhibited a better agreement with the Monte Carlo results for the 2×2 cm^2^ and the Batho, Modified Batho, equivalent TAR and AAA methods for the 10×10 cm^2^ irradiation field.

At the first interface (soft tissue/bone) no difference was exhibited between the algorithms. However, inside the bone, it is also possible to observe that the best choice is not to use heterogeneity correction algorithm.

The characteristics exhibited by PDD curves obtained using Monte Carlo simulation can be explained based on the lateral electronic disequilibrium effect that is more important for small fields. The PDD curves determined by the Eclipse planning system differ from the Monte Carlo PDD curves, because the heterogeneity correction algorithms considered in this work do not take into account the transport of electrons and therefore they are not able to evaluate the lateral electronic disequilibrium. They admit that the deposition of energy in the path is local, or either, all the energy transferred by photons to electrons inside of the irradiation field will be deposited inside the irradiation field. When the distance from a point of interest within a field to the field edge is equal to or smaller than the Compton range for given energy, the Compton interaction produces an electron that can transfer its energy to a point outside the radiation field. When the range of the Compton electrons generated is half the size of the irradiation field, any interaction will produce an electron that can transfer its energy to a point outside the radiation field. Therefore, even those interactions occurring on the central beam axis generate electrons that are not replaced by other electrons generated elsewhere in field and therefore, the electronic equilibrium is lost.

**Figure 5:**
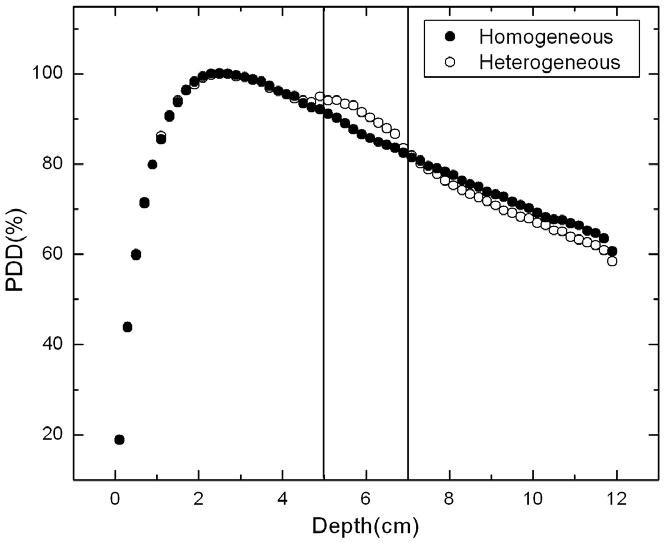
Comparison between PDD curves for heterogeneous and homogeneous materials. The curves were obtained by Monte Carlo simulation for heterogeneous (soft tissue and bony tissue) and homogeneous (soft tissue) materials, considering a 2×2 cm^2^ field size.

It is interesting to observe that bone has a large effect on the central axis dose of small photon beams. The dose to the bone is increased while the dose beyond the bone is decreased as shown in the figure 5 for 2×2 cm^2^ irradiation field. These effects may have clinical implications in the delivery of small fields in IMRT or radiosurgery.

**Figure 6:**
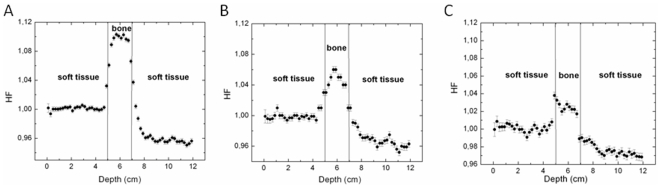
Heterogeneity factor (HF). HF calculated with EGSnrc Monte Carlo code for the (a) 1×1, (b) 2×2 and (c) 10×10 cm^2^ field sizes.

To quantify the magnitude of this effect a heterogeneity factor (HF) is defined as the ratio between the values of the PDD considering the bone presence and the homogeneous material. Figure 6 show the CF for 1×1, 2×2 and 10×10 cm^2^ irradiation fields.

Figure 6 shows that the PDD, or the absorbed dose, is increased at about 10, 5 and 3% respectively to 1×1, 2×2 and 10×10 cm^2^ field sizes due to lateral electronic disequilibrium effect that is more important for small fields. The correction factor increases inversely with irradiation field size because this effect in soft tissue is more significant than in the bone.

### Conclusion

Although the results presented in this work have been obtained for a particular geometry, some interesting conclusions can be obtained:

The head phantom made of acrylic and PVC was tested through the comparison between PDD curves generated by the EGSnrc Monte Carlo code for bone and PVC. The results confirm that, for 2×2 and 10×10 cm^2^ field sizes and 15 MV X-rays beams, PVC can be used as a substitute material for cortical bone.

The PDD curve obtained in water with the ionization chamber was reproduced by EGSnrc simulation, validating the 15 MV spectrum obtained from the literature [20] and the algorithm utilized by EGSnrc.

If the bony heterogeneity is not taken into account, differences of 7 and 4% can be found in PDD planning to 2×2 and 10×10 cm^2^ field sizes, respectively, at soft tissue after this heterogeneity.

Applying the Batho method for correction at the same region, differences of 4% are found in relation to Monte-Carlo calculations. This method is not recommended to adjust the dose after a bony heterogeneity for small field sizes. These differences may conduct an under dosage to the adjacent tissue of the bone. The Equivalent TAR method presented better agreement with the Monte Carlo results for the 2×2 cm^2^ and Batho, Modified Batho, equivalent TAR and AAA methods for the 10×10 cm^2^ field size.

Inside small field sizes, for instance 1×1 and 2×2 cm^2^, that have a typical area of a beam segment used in new radiotherapy technologies, such as the intensity modulated radiation therapy, it takes place the lateral electronic disequilibrium, which is not correctly modelled by the correction algorithms used by the planning system. This fact takes place because the range of the electrons generated inside the irradiation field is higher than the field size. Therefore, many electrons deposit their energy outside the field area, resulting the reduction of the PDD.

Inside the bone, no correction method presents a good performance, since none of them considers the electron transport. The methods Batho, Modified Batho and equivalent TAR present even worse PDD results when compared with no correction PDD results.

The presence of a 2 cm bony heterogeneity (PVC plates) between the acrylic plates that simulate the soft tissues alters the absorbed dose profile inside the heterogeneity by a factor of 10, 5 and 3% for 1×1, 2×2 and 10×10 cm^2^ field sizes, respectively. These dose increments are due to the raise of Compton scattering, the predominant interaction inside the bony material for the energy range considered, since this material processes an electronic density higher than that of acrylic. Such differences are not adequate in radiation therapy considering the total treatment uncertainty, 5%, in delivering the absorbed dose to the target volume, as recommended by publications[25], [26] are still more restrictive and recommend a maximum uncertainty between 3 and 3.5%, considering one standard deviation.
